# Biotic factors influencing the unexpected distribution of a Humboldt marten (*Martes caurina humboldtensis*) population in a young coastal forest

**DOI:** 10.1371/journal.pone.0214653

**Published:** 2019-05-01

**Authors:** Charlotte E. Eriksson, Katie M. Moriarty, Mark A. Linnell, Taal Levi

**Affiliations:** 1 Department of Fisheries and Wildlife, Oregon State University, Corvallis, OR, United States of America; 2 Pacific Northwest Research Station, USDA Forest Service, Olympia, WA, United States of America; 3 Department of Forest Engineering, Resources, and Management, Oregon State University, Corvallis, OR, United States of America; Smithsonian Conservation Biology Institute, UNITED STATES

## Abstract

Pacific martens (*Martes caurina*) are often associated with mature forests with complex structure for denning, resting, and efficient hunting. Nonetheless, a small isolated population of the Humboldt subspecies of Pacific martens (*Martes caurina humboldtensis*) occupies a narrow strip of young, coastal forest (< 70 years old) but not inland mature forest in the central Oregon Coast Range. We examined factors contributing to this unexpected distribution of martens by 1) analyzing marten diets using DNA metabarcoding to assess 90 scats, 2) using camera traps to assess differences in the relative abundances of prey, competitors, and predators across a coastal to inland gradient of vegetation types, and 3) quantifying differences in extent of fruit-producing shrubs and vegetation structure within vegetation types. Diets of martens were diverse (12, 10, and 3 species of birds, mammals, and amphibians respectively), and most fall and winter scats contained fruit. Voles, mice, and varied thrushes (*Ixoreus naevius*) were dominant prey items. Voles, mice, and most birds, but not varied thrushes, were more commonly observed in the coastal shrub-dominated forest than in inland forest. The coastal shrub-dominated forest had the highest diversity of vertebrates and potential prey overall. Bobcats (*Lynx rufus*), a key potential predator, were more commonly detected in inland forest. Of potential competitors, western spotted skunks (*Spilogale gracilis*) were more commonly detected in inland forest, with gray foxes (*Urocyon cinereoargenteus*) and raccoons (*Procyon lotor*) detected almost exclusively in coastal forests. Vegetation in coastal forests appears to provide, at least seasonally, more prey and fruit, and more overhead shrub cover compared with inland forest. Remaining plausible hypotheses for the restricted distribution of marten to coastal forests include increased prey, fruit, and overhead cover, and reduced predation risk from bobcats.

## Introduction

Understanding the drivers that shape a species’ distribution is a fundamental aspect of conservation. These drivers may be abiotic conditions including climate, altitude, and elevation [1–4] or biotic factors such as prey availability, competition and the influence of predators [2,4–6]. Although examples exist with clear causal mechanisms that facilitate presence or recovery of species, commonly we are limited in the availability of natural history data to establish these linkages. A lack of information regarding key prey species, competitors, and predators restricts inferences about whether species interactions influence species’ distribution [7]. This may be particularly relevant for mid-trophic taxa such as small carnivores that are subject to predation but may also be limited by prey availability [8]. Here we focus on potential effects of species interactions on the distribution of a poorly studied small carnivore, the Humboldt marten (*Martes caurina humboldtensis*) subspecies in the Pacific Northwest of North America.

Humboldt martens in the central coast of Oregon were withdrawn from listing consideration under the United States Endangered Species Act in 2015 [9]. The withdrawal concluded that large areas of mature forests present within their historical range provided sufficient habitat for the species to persist [9,10]. After the listing decision, subsequent distributional surveys have suggested that despite extensive older and mature inland forests within the central coast range of Oregon, these forests did not currently support a marten population [11]. Instead, a Humboldt marten population appears isolated to a narrow strip of young coastal forests (< 70 years old) growing on sandy soils on the margin of the Pacific Ocean [11], contrasting prevailing observations that martens require old (e.g., > 200 years) structurally complex forests [12]. The population estimate for this remnant marten population was 41–87 individuals living at unusually high density [13]. Evidence that the current populations of Humboldt martens appear small and isolated have contributed to a recent proposal to reconsider for listing the coastal Distinct Population Segment as Threatened [14]; however, little information is available describing predicted habitat and distribution of these coastal martens.

North American marten (*M*. *caurina*, *M*. *americana*) densities have been correlated with the abundance of prey species such as voles [15], mice, and squirrels [16]. In comparison to other carnivores, martens have high metabolism and limited fat reserves [17]. A typical marten must consume approximately 25% of its body weight daily, the equivalent of 7 red-backed voles (25 g each) (*Myodes* spp.), a common prey item across the range of these species [18]. Due to their relatively high metabolic rate, martens could be limited by bottom-up resource availability, suggesting that differences in prey availability could drive the distribution of marten. However, being a small-sized carnivore, martens must balance their high nutritional requirements with predation risk by larger mammalian carnivores such as bobcats (*Lynx rufus*), coyotes (*Canis latrans*), fishers (*Pekania pennanti*), and avian predators [19,20]. Martens are often associated with dense overstory cover and forests with structural complexity that are thought to reduce predation risk [21]. Differences in structural complexity and/or the abundance of their predators are thus plausible hypotheses to at least partially explain the observed Humboldt marten distribution. Similarly, other carnivores of similar body size and diet composition, such as gray fox (*Urocyon cinereoargenteus*), raccoon (*Procyon lotor*), and western spotted skunk (*Spilogale gracilis*) could also play a role in Humboldt marten distribution in coastal Oregon.

Structurally-complex mature forests, typified by logs, legacy trees, and snags >200 years old [22], are thought to fulfill the life-history requirements of North American martens. Such forests provide thermally efficient resting and denning sites [23,24], increased prey availability [16,25], foraging success [26], and predator protection. It is unclear whether and how the young coastal forest in Central Oregon provides for the life history requirements of Humboldt martens (henceforth, martens), but better understanding of the ecological context may be critical to marten conservation.

The apparent contradiction between habitat associations based on forest age, and a broad lack of natural history information about this marten population motivated our study. Our objectives were to assess factors contributing to the observed restricted distribution of martens to coastal forests compared to inland forest by 1) quantifying the diet of martens to determine nutritional resources consumed in the coastal forests, 2) quantifying differences in the relative abundance of prey, predators, or competitors, and 3) assessing differences in fruit availability and vegetation structure. We quantified marten diet with DNA metabarcoding of scats followed by mechanical sorting of a subset of scats to assess seed and invertebrate composition. Because it was unclear *a priori* whether a particular vegetation type might support key prey, predator or competitor species influencing the persistence of martens, we used grids of camera traps to characterize relative abundance of these species across our vegetation type gradient. Finally, we conducted vegetation surveys at all camera stations to quantify ground cover, shrub, and overstory characteristics, and cover of fruit-producing shrubs.

## Materials and methods

### Study area

**Fig 1:**
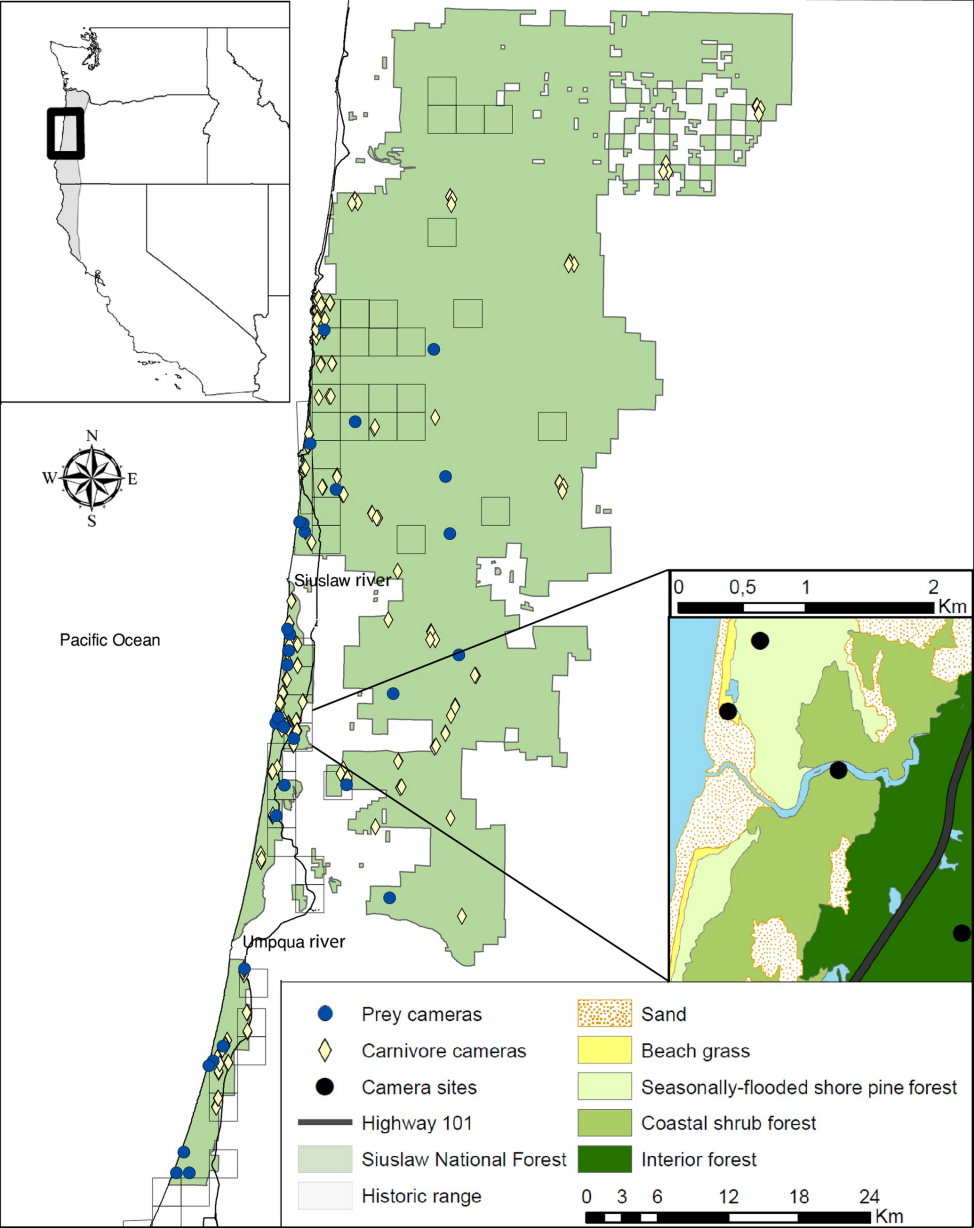
Map of Humboldt marten (*Martes caurina humboldtensis*) study area and survey locations on the central coast, Oregon, USA. We collected data on potential marten prey (*n* = 31 grids with 20 baited cameras per grid, 620 camera stations (circles). For potential competitors and predators, we used data from 259 sample units (*n* = 843 camera stations including the cameras on the grids) in forest east of the coastal highway (*n* = 86 baited, 55 unbaited trail camera stations) and coastal forests (*n* = 74 baited, 44 unbaited trail) (diamonds). Six marten detections (*n* = 4 genetically verified scats, 2 remote camera photographs) were within 500–700 m from the Pacific Ocean, with the remainder (*n* = 86 genetically verified scats, 5876 remote camera photographs) within the Oregon Dunes National Recreation Area. Our inset (upper left) shows the Humboldt marten range (grey) and study area location where the inset on the lower right depicts an example of the prey grid locations amongst the 4 vegetation types.

Our study occurred in the Siuslaw National Forest on the central Oregon coast, United States, with most coastal forests located in the Oregon Dunes National Recreation Area, hereafter Oregon Dunes (43°42’ N, 124°10’W) (Fig 1). This region had a mild Pacific maritime climate with mean minimum and maximum temperatures of 10.1°C and 20.3°C in summer and 3.2°C and 10.2°C in winter. Total rainfall per year was approximately 1800 mm (Western Regional Climate Center 2017; https://wrcc.dri.edu/).

We stratified the study area into 4 vegetation types (listed from coast to inland) based on species dominance adapted from [27]: 1) foredune beach grass, hereafter “beach grass” 2) shore pine (*Pinus contorta*)/slough sedge (*Carex obnupta*) seasonally flooded forests located in the deflation plains, hereafter “seasonally-flooded shore pine forest”, 3) shore pine-sitka spruce (*Picea sitchensis*) forest with a dense ericaceous understory dominated by evergreen huckleberry (*Vaccinium ovatum*) and salal (*Gaultheria shallon*), hereafter “coastal shrub forest” and 4) Douglas-fir (*Pseudotsuga menziesii*) and sitka spruce dominated forests, inland of the coastal zone, hereafter “interior forest”. The 4 vegetation types were located within close proximity to each other, occasionally with all 4 types spaced within 2.5 km from the Pacific ocean (Fig 1).

### Marten diet

We quantified diet from marten scats using DNA metabarcoding. We acquired scats in 3 ways: using scent detection dog teams (2015–2017), by observers opportunistically when trapping martens (2015–2016), and while identifying locations where martens rested (Winter 2016), during a separate study [28] (S1 Document). Detection dog surveys either saturated accessible young coastal forests in the central Oregon coast or were placed using a stratified random design in the interior forest in 3 x 3 km sample units with searches > 6 hours (see [29] for methods).

We dried scats in the field and processed them in the lab. We extracted DNA for species and diet composition in a laboratory dedicated to processing degraded DNA using the QIAamp DNA Stool Mini Kit (Qiagen, USA). We included an extraction blank with every batch of extractions as a negative control to monitor for cross-contamination. We used slightly modified universal vertebrate primers 12SV5F (TTAGATACCCCACTATGC) and 12SV5R (YAGAACAGGCTCCTCTAG) to amplify the ribosomal mitochondrial 12S gene region (adapted from [30]). We performed 3 PCR replicates per scat with Qiagen Multiplex PCR Kit (Qiagen, USA). Each reaction was amplified with identical unique 8 base pair tags on the 5’ end of the forward and reverse primer to identify individual scat samples after pooling and to prevent false assignment due to tag jumping [31]. PCR reactions were carried out in a volume of 20 μL using the following reagent mixtures: 10 μL Qiagen Multiplex PCR Master Mix (Qiagen, USA), 0.4 μL of each primer for a final primer concentration of 200 nM, 0.2 μL bovine serum albumin (BSA), 6.2 μL of water, and 1 μL final DNA extract elution (including extraction blanks and PCR no template controls). Following a 15 minutes initial denaturation at 95°C, the cycling conditions were: 40 cycles of 94°C for 30 seconds, 58°C for 90 seconds, 72°C for 90 seconds, and a final extension at 72°C for 10 minutes. We normalized and pooled the PCR products and used NEBNext Ultra II Library Prep Kit (New England BioLabs, USA) to adapt the library pools into Illumina sequencing libraries (Illumina Inc, USA). Libraries were purified using the Silica Bead method (Aline Biosciences, USA) and sent for 150 bp paired-end sequencing on the Illumina HiSeq 3000 at the Center for Genome Research and Biocomputing, Oregon State University. The raw reads were paired using *PEAR* [32] and demultiplexed based on the unique 8bp-index sequences using a custom shell script. Unique reads from each sample replicate were counted and taxonomically assigned using BLAST (www.ncbi.nlm.nih.gov/blast), against all 12S vertebrate sequences in Genbank and against a custom 12S library created for vertebrates not present in Genbank (S2 Document). We used the negative controls to set filtering read thresholds and assigned species if present in at least 2 out of the 3 replicates.

We manually sorted a random subset of scats from each season (~40%) to determine the presence of seeds and invertebrates. Each scat was soaked over a fine mesh, dried and then mechanically sorted using a dissecting microscope. We identified seeds to species and invertebrates to order. We recorded all dietary items using relative frequency of occurrence (number of occurrences of each species/number scats) by season.

### Relative abundance of potential prey species

To inform availability of marten prey, we quantified an index of relative prey abundance using grids of remote cameras randomly placed within each of our 4 vegetation types during October to December 2015 (Fig 1). We defined prey as vertebrates that we detected in marten diet, or vertebrates morphologically similar to prey previously recorded in marten diet studies [33] and mountain beaver (*Aplodontia rufa*), which we included as potential prey due to its locally endemic distribution and body mass within the range of marten prey items [34]. We followed previous research that used track plates [35] to estimate an index of small mammal abundance, but used remote cameras rather than tracks to identify occurrence. We recorded occurrence of vertebrates within prey camera grids consisting of 20 remote cameras (Bushnell Aggressor, model 119776, Overland Park, MI) spaced 30 m apart in 2 parallel transects. A trap spacing of 20 m has been recommended for cricetid rodents for mark-recapture density estimates [36]. However, we chose 30 m to balance detections of cricetid rodents with species that have larger home ranges (e.g. sciurids). We placed cameras 20 cm high, 1.5 m from bait. Bait consisted of 15 grams each of sunflower seeds, peanut butter, oats, commercial rabbit food and strawberry jam placed within a pocket (20 x 10 cm) of fine metal mesh nailed to the ground. The mesh ensured that bait was released slowly such that it was present for the duration of camera deployment. Remote cameras monitored bait for 7 to 15 consecutive days and were programmed to take 3 photos per trigger with 1 min delay between triggers. We defined our index of relative abundance as number of cameras detecting a species on a camera grid within a 7-day period for all taxa, except deer mouse (*Peromyscus maniculatus*), which was assessed within a two-day window to avoid detection saturation at all cameras. Our purpose here was to estimate relative abundances within species among 4 vegetation types. We also note that our approach cannot be used to compare relative abundances across species, due to differences in movement, foraging, and spacing patterns that influence probabilities of detection.

For each camera, we assigned a 1 if a species was detected at least once and 0 if it was not detected within 7 days (or 2 days for deer mice). The number of cameras per grid detecting a given species relative to the number of functional cameras (20, unless a camera malfunctioned) was treated as a binomial response variable (*K* successes in *N* trials) in a generalized linear mixed-effects model (R function *glmer* from the lme4 package [37]). We included a random effect at the grid level to account for overdispersion as recommended by [38]. For each species, we included a factor level for each vegetation type and used Tukey’s honest significant difference (HSD) to test for differences in relative abundance across all pairwise combinations of vegetation types (see R code in S3 Document). We excluded species detected in fewer than 3 grids due to inadequate sample sizes.

We examined trends in species diversity among vegetation types by calculating the reciprocal of Simpson’s index per grid and averaging it across vegetation types [39]. We assessed the difference in diversity in 3 categories: total vertebrate diversity (all vertebrate species detected), potential marten prey diversity (small mammal and bird species), and carnivore species diversity. As voles and shrews were not identified to species, diversity indices for all vertebrate species and prey species are potentially biased lower.

### Relative abundance of competitors and predators

We estimated an index of abundance of carnivores, which we assumed could be predators or competitors of martens. Because carnivore home ranges (> 2 km^2^) could readily overlap the beach grass, seasonally-flooded shore pine forest, and coastal shrub forest vegetation types, we combined them into “coastal forests”. Thus, for our carnivore analysis we compared coastal forests with interior forest. We used 4 camera data sources to create our carnivore abundance index: 1) We paired 2 camera stations with each prey grid > 75 m away and separated by > 50 m following the protocol in [29]. We baited 1 station with chicken and an olfactory lure, hereafter a “baited” station (Gusto, Minnesota Trapline Products, USA), and 1 was left unbaited on a trail, hereafter “unbaited trail” station. Cameras (*n* = 40 baited, 40 unbaited trail) were deployed for 7–15 days. 2) We used data within our study area from [29] (*n* = 79 baited, 50 unbaited trail cameras) deployed May–October 2015. These cameras were placed in areas 5–50 km from historic marten detections using a stratified random sampling design to ensure even distribution of samples in early to mature forest age classes. We deployed cameras for a minimum of 21 days, baited with Gusto and chicken or cat food (for more details see [29]. 3) We used camera data from our marten surveys in the coastal forests (*n* = 9 baited, 10 trail), which were deployed for an average of 35 days October 2015 –February 2016, and were baited with chicken, cat food, and strawberry jam [13]. 4) Because our camera grids designed to identify prey frequently also detected carnivores, we pooled observations from all cameras on the grid, which we then treated as a single baited camera station (*n* = 31 camera grids, 620 camera stations). Combined, we included data from 259 sample units (*n* = 843 camera stations including the camera stations on the grids). For analyses, our sample sizes were similar between the interior (*n* = 86 baited, 55 unbaited trail camera stations) and coastal forests (*n* = 74 baited, 44 unbaited trail) May 2015 –October 2016 (Fig 1). We excluded carnivore species that were detected with fewer than 5 cameras from further analysis due to inadequate sample sizes.

We used the camera trapping rate as an index of abundance, but to avoid over-weighting repeat visits within a short time interval, we treated each day, rather than photo, with a species detection as an encounter out of *N* total potential encounters, where *N* was the number of sampling days. For each carnivore or potential competitor, we used a binomial generalized linear mixed-effects model (R function *glmer* from the lme4 package; [37]) with the number of days detecting a species relative to the number of monitoring days per camera as the binomial dependent variable. We included forest (coastal vs interior) as a covariate to test for differences in relative abundance between the forests, and a random effect of sample unit. To account for differences in probability of detection by camera set type, we included binary factors (fixed effects) for unbaited trail camera vs. baited camera, and prey camera grid vs. single camera. By accounting for the assumed higher probability of detection on the prey camera grid, or when using bait, we were able to incorporate all available data in our analyses. If we found significant differences in the probability of carnivore detection between the coastal forests and interior forest, we assessed whether significance (*p*-value) was inflated by pseudoreplication due to spatial autocorrelation using a Moran's I test on the residuals [40]. If the Moran’s I test was significant, we included a spatial lag autocovariate term in the model and report the Moran’s I *p*-value in the more inclusive model [40].

All camera surveys followed protocols approved by the USDA Forest Service, Pacific Northwest Research Station, and surveys were conducted under Oregon Department of Fish and Wildlife Scientific Take Permits (119–15, 128–16).

### Vegetation surveys

To compare vegetation among our 4 vegetation types, we performed ocular vegetation surveys at each camera station within the prey camera grids. We estimated percentage cover of the canopy, shrub and ground layers, shrub height (m), tree height (m), and tree diameter (cm) at breast height within a 10 m radius circular plot centered on the camera. All vegetation surveys were performed by 1 observer, and we calculated averages per vegetation community using Kruskal-Wallis tests and adjusted for multiple comparisons using the False Discovery Rate method [41]. We estimated approximate forest age of the camera grids using gradient nearest-neighbor maps (2012 version accessed from: https://lemma.forestry.oregonstate.edu/data). Gradient nearest-neighbor techniques combine data from on the ground vegetation plots from the Forest Inventory and Analysis National Program with remotely sensed spectral data to infer forest quality and characteristics in areas without vegetation plots [42].

## Results

### Diet analysis

We collected 108 scats during 3 seasons, of which 98 were genetically confirmed to be from marten, 5 gray fox, 1 raccoon, 1 bobcat and 3 scat samples contained only prey DNA. Four marten scats were located within the interior forest approximately 200–300 m east of the coastal forests where the remainder of the scats were found.

Eight marten scats yielded marten DNA exclusively, leaving 90 scats for diet analysis. We identified 25 vertebrate taxa (Table 1), 3 plant species, and 5 insect orders. Martens fed extensively on berries during fall (100% of scats) and winter (86% of scats) (Table 2). Invertebrates were eaten in small amounts across all seasons and comprised mainly of beetles (Coleoptera), bees and ants (Hymenoptera), but were not the dominant component of any scats (Table 2). Mammals featured consistently in the diet across spring, fall and winter with voles being the most frequent prey group (55%; Table 1). Western red-backed vole (*Myodes californicus*) and deer mouse were common food items (32.2% and 17.8%, respectively). Eleven percent of the scats contained red tree vole (*Arborimus longicaudus*), a strictly arboreal species. In addition, white-footed vole (*Arborimus albipes*), a little known arboreal species [43], occurred in 13.3% of the scats. We documented birds more frequently in scats from winter (88.0%) compared to fall (41.9%; *β* = -3.24, *P* < 0.01) and spring (38.2%; *β* = -3.48, *P* < 0.01). Birds occurred on average in 53.3% of marten scats with varied thrush being the overall most frequently consumed species (45.6%) (Table 1).

**Table 1:** Percent occurrence of vertebrate food items in spring, fall and winter of Humboldt martens (*Martes caurina humboldtensis*) in the central Oregon coast from April 2015—March 2016. Data were based on 90 genetically confirmed marten scats collected using scent detection dog surveys (*n* = 30), live-trapping and from known marten rest structures (*n* = 60). The samples were grouped per season (spring: March–May 34 scats, fall: September–November 31 scats, and winter: December–February 25 scats). We quantified vertebrate diet composition using DNA metabarcoding.

		Spring (%) Fall (%) Winter (%) Total (%)			
Prey		*(n* = 34)	(*n* = 31)	(*n* = 25)	(*n* = 90)
Sample size		34	31	25	90
**Birds**		38.2	41.9	88	53.3
*Corvidae*	Steller's jay (*Cyanocitta stelleri*)	2.9	0	0	1.1
*Parulidae*	Yellow-rumped warbler (*Setophaga*	0	3.2	8	3.3
	*coronata*)				
				
*Phasianidae*	Ruffed grouse (*Bonasa umbellus*)	2.9	0	0	1.1
*Picidae*	Northern flicker (*Colaptes auratus*)	0	0	8	2.2
*Rallidae*	American coot (*Fulica americana*)	0	3.2	0	1.1
*Regulidae*	Golden-crowned kinglet (*Regulus satrapa*)	0	6.5	12	5.6
*Sylviidae*	Wrentit (*Chamaea fasciata*)	5.9	6.5	0	4.4
*Throchilidae*	Rufous hummingbird (*Selasphorus rufus*)	2.9	0	0	1.1
*Troglodytidae*	Pacific wren (*Troglodytes pacificus*)	0	0	4	1.1
*Turdidae*	American robin (*Turdus migratorius*)	0	0	8	2.2
	Swainson's thrush (*Catharus ustulatus*)	0	0	4	1.1
	Varied thrush (*Ixoreus naevius*)	26.5	35.5	84	45.6
**Mammals**		70.6	87.1	80	80
*Cervidae*	Black-tailed deer *(Odocoileus hemionus)*	0	35.5	0	12.2
*Cricetidae*	White-footed vole (*Arborimus albipes*)	11.8	22.6	4	13.3
	Red tree vole (*Arborimus longicaudus*)	29.4	0	0	11.1
	Western red-backed vole (*Myodes*	26.5	22.6	52	32.2
	*californicus*)				
				
	Creeping vole (*Microtus oregoni*)	0	3.2	20	6.7
	Deer mouse (*Peromyscus maniculatus*)	3.4	25.8	28	17.8
*Leporidae*	Brush rabbit (*Sylvilagus bachmani*)	0	0	4	1.1
*Sciuridae*	Townsend's chipmunk (*Tamias townsendii*)	0	3.2	0	2.2
	Douglas squirrel (*Tamiasciurus douglasii*)	11.8	0	0	4.4
*Soricidae*	Trowbridge's shrew (*Sorex trowbridgii*)	5.9	0	0	2.2
**Amphibians**		8.9	3.2	4	5.6
*Ambystomatidae*	Coastal giant salamander (*Dicamptodon*	2.9	0	0	1.1
	*tenebrosus*)				
*Hylidae*	Pacific chorus frog (*Pseudacris regilla*)	0	3.2	4	2.2
*Plethodontidae*	Wandering salamander (*Aneides vagrans*)	5.9	0	0	2.2

**Table 2:** Percent occurrence of berries and invertebrates from a subsample of Humboldt marten (*Martes caurina humboldtensis*) scats collected in the central Oregon coast April 2015 –March 2016. We manually sorted a random subset of marten scats (*n* = 35) and examined contents using a dissecting microscope because we were unable to detect berries and invertebrates using our vertebrate primers during DNA metabarcoding. We grouped samples by season (spring: March–May, fall: September–November, and winter: December–February).

Food group		Spring (%) *n* = 11	Fall (%) *n* = 10	Winter (%) *n* = 14	Total (%) *n* = 35
**Fruit**		0	100	85.7	62.9
*Ericaceae*	Evergreen huckleberry *(Vaccinium ovatum)*	0	90	85.7	54.5
*Rosaceae*	*Rubus* spp.	0	20	53.8	50
Unknown spp.	* *	0	0	35.7	35.7
**Invertebrates**		18.2	45.5	50	38.9
	Coleoptera	9	9	14	18.2
	Diptera	0	0	7	4.5
	Hymenoptera	0	9	28.6	22.7
	Oribatida	0	9	0	4.5
	Trichoptera	0	9	0	4.5
	Unknown insect	9	9	0	9

### Relative abundance indices–potential prey

**Fig 2:**
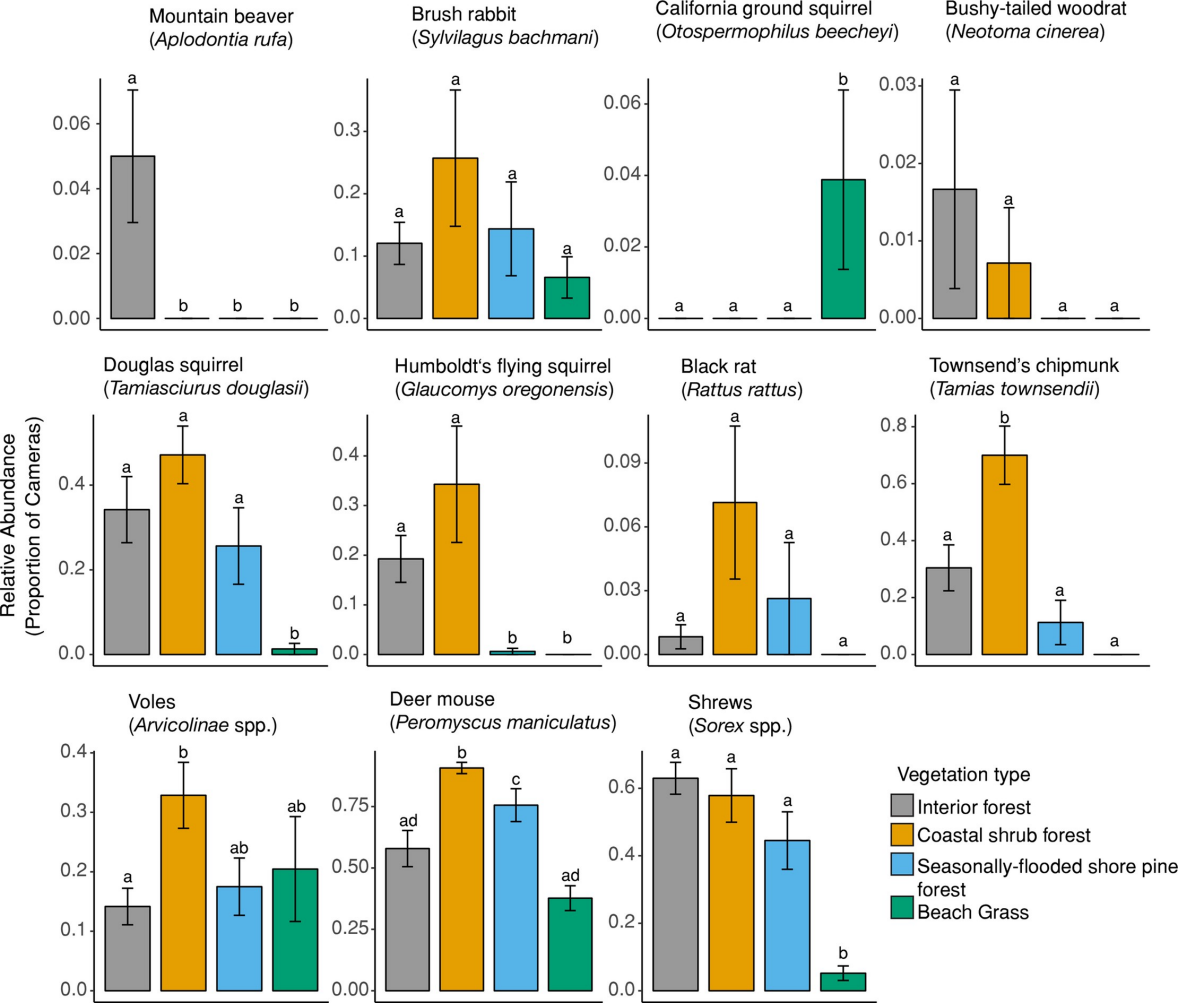
Relative abundance ($\bar{x}$ *± SE*) of small mammals by vegetation type in the central Oregon coast during October–December 2015. We defined relative abundance as the proportion of cameras that detected a species per vegetation community. We deployed 620 camera stations across 31 grids in 4 vegetation types (interior forest *n* = 12 grids, coastal shrub forest *n* = 7 grids, seasonally-flooded shore pine forest *n* = 8 grids, and beach grass *n* = 4 grids). Species plots are arranged based on average body mass from heavier to lighter (left to right). Different letters denote significant difference (*P* ≤ 0.05) among vegetation types after Tukey’s HSD adjustment for multiple comparisons. Plots without any letters indicate that there was no significant difference between any vegetation types.

We deployed 620 camera stations across 31 grids in 4 vegetation types to quantify relative prey abundance (interior forest *n* = 12 grids, coastal shrub forest *n* = 7 grids, seasonally-flooded shore pine forest *n* = 8 grids, and beach grass *n* = 4 grids) (Fig 1). Two grids had 1 camera malfunction each, and 1 grid had 3, leaving 615 camera stations. We detected 34 potential prey species (16 small mammal and 18 bird species). Because shrews and voles were only identified to genus (*Sorex*) and subfamily (*Arvicolinae*), respectively, the actual number of small mammal species is likely higher because there are 5 shrew and 6 vole species present in this region [34]. Deer mice were documented at every grid across all vegetation types but were less abundant in interior forest compared to coastal shrub forest (*β* = -4.24, *P* < 0.001). Similarly, voles were observed in all vegetation types but were less abundant in interior forest compared to other vegetation types (*β* = -2.94, *P* = 0.02). In contrast, mountain beaver and California ground squirrel (*Otospermophilus beecheyi*) were only detected in interior forest and beach grass, respectively. The other squirrel species were observed in all forested vegetation types with Townsend’s chipmunk (*Tamias townsendii*) being less abundant in the interior forest (*β* = -2.99, *P* = 0.01) (Fig 2).

**Fig 3:**
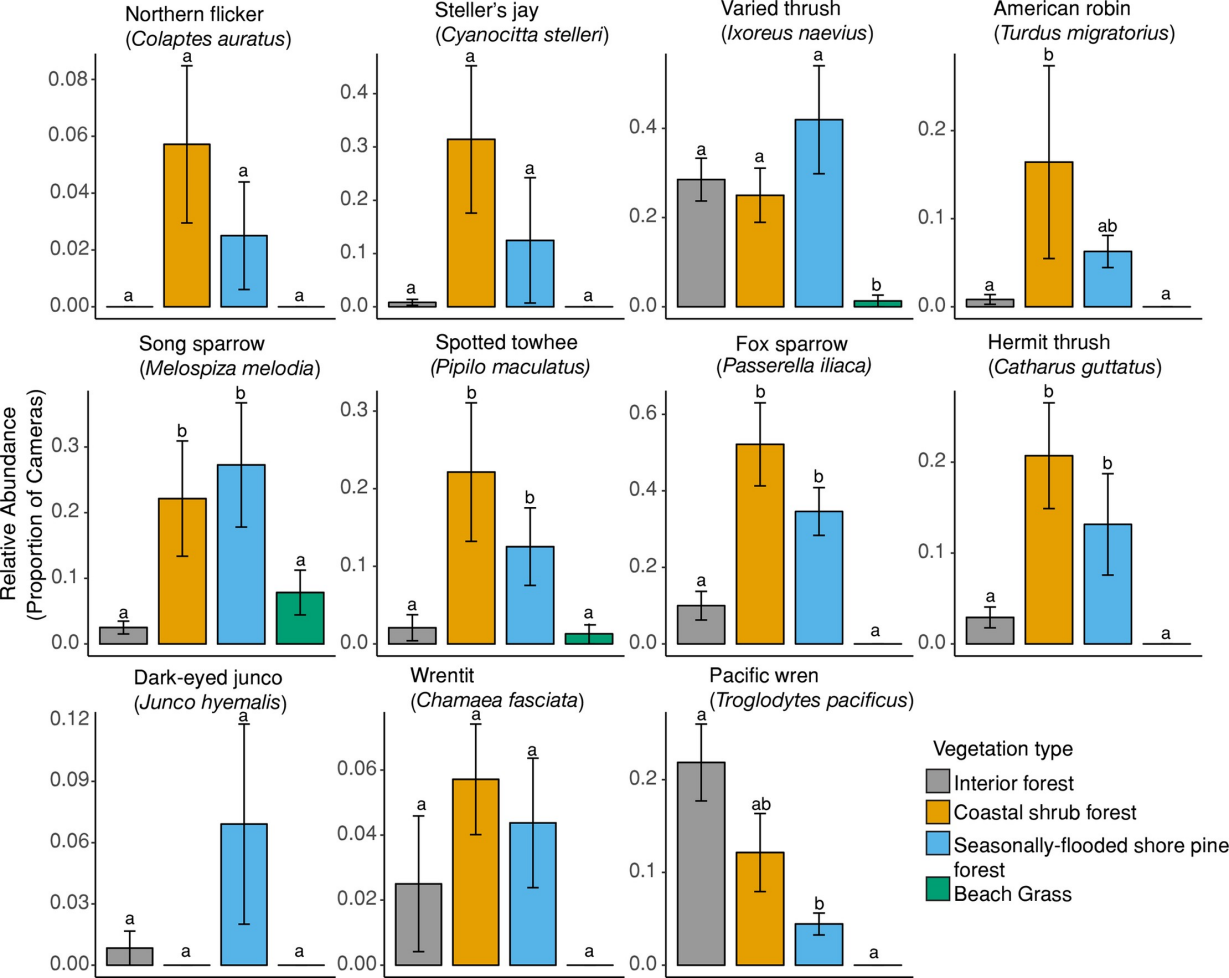
Relative abundance ($\bar{x}$ *± SE*) of birds per vegetation type in the central Oregon coast during October–December 2015. We defined relative abundance as the proportion of cameras that detected a species per vegetation type and calculated standard error of the difference between the vegetation types. We deployed 620 camera stations across 31 grids with 20 cameras each (interior forest *n* = 12 grids, coastal shrub forest *n* = 7 grids, seasonally-flooded shore pine forest *n* = 8 grids, and beach grass *n* = 4 grids). Species plots are arranged based on average body mass from heavier to lighter (left to right). Different letters denote significant difference (*P* ≤ 0.05) among vegetation types after Tukey’s HSD adjustment for multiple comparisons. Plots without any letter indicate that there was no significant difference between any vegetation types.

The most commonly detected bird species were varied thrush, fox sparrow (*Passerella iliaca*), song sparrow (*Melospiza melodia*), and spotted towhee (*Pipilo maculatus*, Fig 3). Five out of 11 bird species were detected significantly more in the coastal shrub forest or seasonally-flooded shore pine forest compared to interior forest (Fig 3). See S1 Table for a complete list of all potential prey species detected and S1 Fig for photograph examples.

### Relative abundance indices–potential predators and competitors

**Fig 4:**
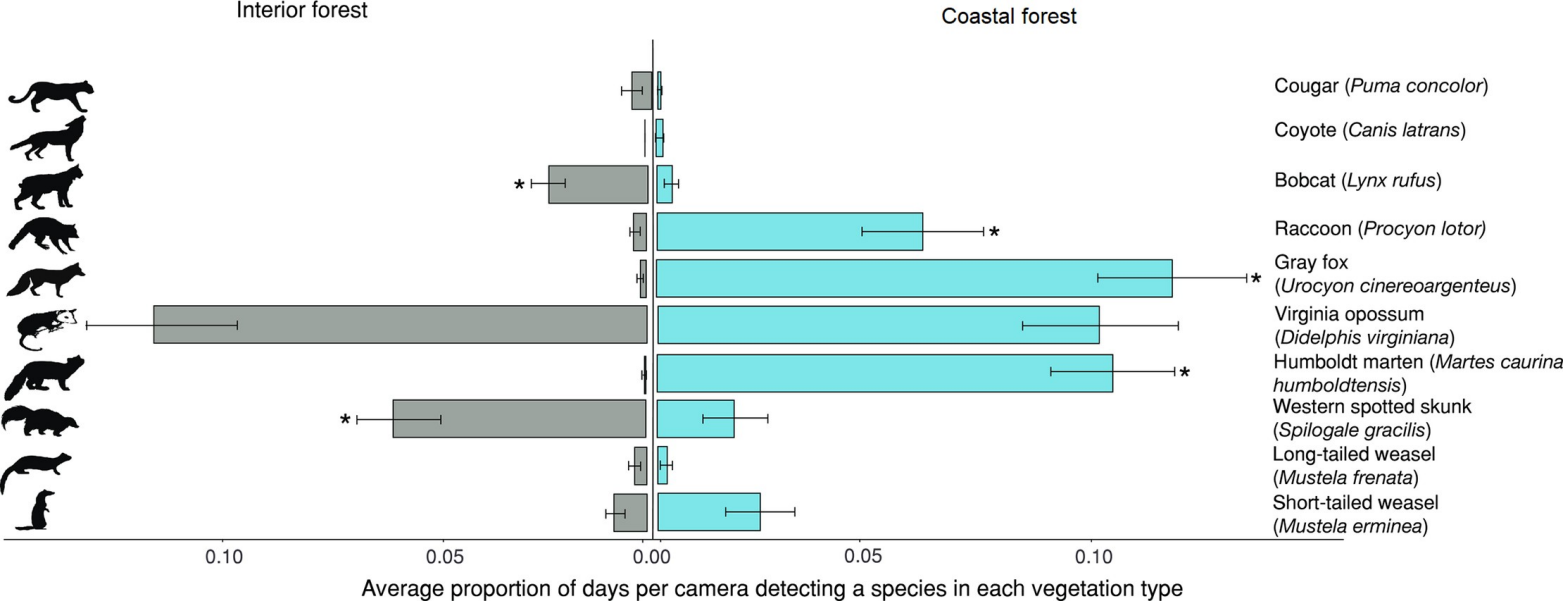
**Relative abundance defined as the average proportion of days per camera detecting a species ($\bar{x}$*± SE*) of carnivores and potential competitors in the interior forest (on the left) and coastal forests (on the right) during an investigation of Humboldt marten (*Martes caurina humboldtensis*) occurrence in the central Oregon coast.** We used 843 cameras combined into 259 spatially distinct sample units (*n* = 160 baited sample units, including the 31 prey camera grids with 20 cameras each) and 99 unbaited camera stations). Species bars are arranged from heavier to lighter species (top to bottom). Stars indicate significant difference in relative abundance between the interior forest and the coastal forests.

Composition and relative abundance of carnivore species varied between coastal forests and interior forest (Fig 4). Martens (*β* = -5.93, *P* < 0.001), raccoons (*Procyon lotor*) (*β* = -2.52, *P* < 0.001), and gray foxes (*Urocyon cinereoargenteus*) (*β* = -4.47, *P* < 0.001) had significantly fewer detections in interior forest compared to coastal forests. In contrast, bobcats and western spotted skunks had significantly higher detections in the interior forest (*β* = 2.29, *P* < 0.001) and (*β* = 2.24, *P* < 0.001), respectively (Fig 4; Table 3). The prey camera setup with 20 cameras per grid significantly increased detection rates relative to the baited and unbaited trail cameras for all carnivore species except martens, long-tailed weasels (*Mustela frenata*), and cougars (*Puma concolor*) (Table 3). Unbaited trail cameras had significantly higher detection rates of bobcats (*β* = 1.12, *P* < 0.001) compared to baited cameras and we observed the opposite relationship for western spotted skunks (*β* = -0.38, *P* < 0.001) (Table 3). Among the species with detection rates that significantly differed between the vegetation types, we found spatial autocorrelation for bobcats (Moran’s I test, *P* < 0.001), cougars (*P* = 0.03), raccoons (*P* < 0.001) and western spotted skunks (*P* < 0.001) which we then accounted for in our models. After adding a spatial lag autocovariate, no additional spatial autocorrelation was found for bobcats (Moran’s I test, *P* = 0.53), cougars (*P* = 0.80), raccoons (*P* = 0.73), or western spotted skunks (*P* = 0.08) (Table 3). All species detected are included in S1 Table.

**Table 3:** We evaluated potential predator or competitor detections, defined as the proportion of days detecting a species, in relation to two vegetation classes during a Humboldt marten (*Martes caurina humboldtensis*) study in central Oregon.

Species	Estimate	Standard error	p-value
Bobcat (*Lynx rufus*)*			
Interior forest	2.296	0.632	<0.001
Unbaited trail camera	1.118	0.307	<0.001
Prey camera setup	-0.251	0.824	0.761
Spatial lag variable	-0.704	0.221	0.001
Moran's I p-value	0.532		
Cougar (Puma concolor)*			
Interior forest	1.168	1.73	0.499
Unbaited trail camera	0.523	0.698	0.454
Prey camera setup	3.447	1.292	0.007
Moran's I p-value	0.80		
**Humboldt marten (*Martes caurina humboldtensis*)**			
Interior forest	-5.928	1.138	<0.001
Unbaited trail camera	-1.021	0.221	<0.001
Prey camera setup	0.065	0.32	0.839
Gray fox (*Urocyon cinereoargenteus*)*			
Interior forest	-4.471	0.737	<0.001
Unbaited trail camera	-0.313	0.215	0.145
Prey camera setup	1.266	0.317	<0.001
Spatial lag variable	-0.449	0.14	0.001
Moran's I p-value	0.212		
**Long-tailed weasel (*Mustela frenata)***			
Interior forest	-0.534	2.239	0.812
Unbaited trail camera	-0.246	0.952	0.796
Prey camera setup	0.04	1.023	0.969
Raccoon (*Procyon lotor*)*			
Interior forest	-2.522	0.584	<0.001
Unbaited trail camera	0.301	0.313	0.336
Prey camera setup	2.288	0.411	<0.001
Spatial lag variable	-0.777	0.178	<0.001
Moran's I p-value	0.732		
**Short-tailed weasel (*Mustela erminea)***			
Interior forest	-0.309	0.532	0.56
Unbaited trail camera	-0.42	0.614	0.494
Prey camera setup	3.249	0.509	<0.001
**Virginia opossum (*Didelphis virginiana*)**			
Interior forest	-0.009	0.522	0.986
Unbaited trail camera	-0.012	0.142	0.933
Prey camera setup	1.442	0.236	<0.001
Western spotted skunk (*Spilogale gracilis*)*			
Interior forest	2.238	0.521	<0.001
Unbaited trail camera	-0.384	0.174	0.027
Prey camera setup	1.636	0.358	<0.001
Spatial lag variable	-0.294	0.049	<0.001
Moran's I p-value	0.082		

We used generalized linear mixed-effects models to examine the influence of vegetation type (interior forest relative to coastal forests), camera type (unbaited trail cameras compared to baited camera), and survey type (prey camera setup versus carnivore camera setup) for each species. If we found significant difference in abundance between the 2 vegetation types, we assessed whether *p*-values were inflated by pseudoreplication due to spatial autocorrelation using a Moran's I test on the residuals. If present, spatial autocorrelation (indicated by *) was accounted for by adding a spatial lag variable to the model and we report our Moran’s I test on the residuals.

**Fig 5:**
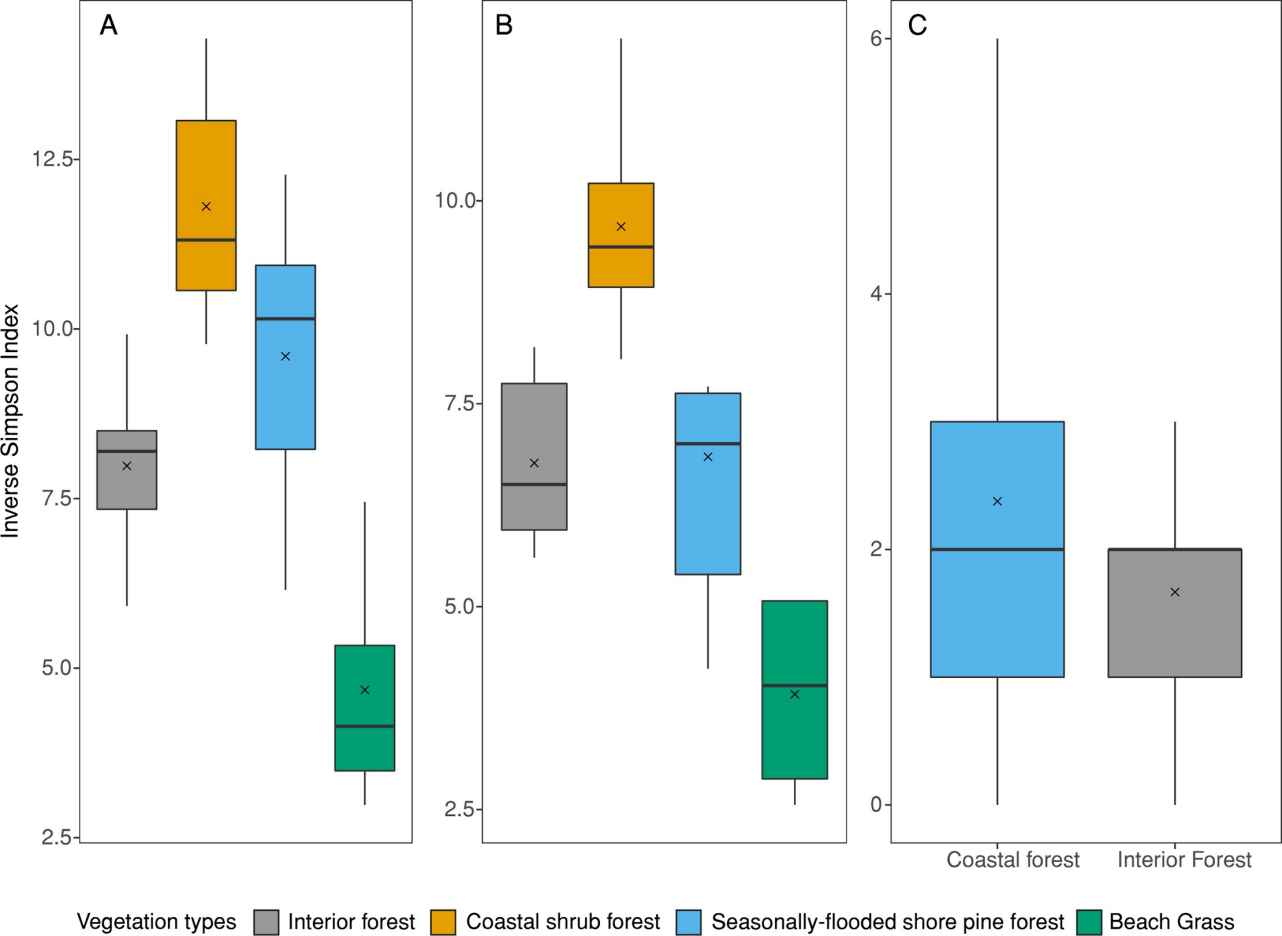
**Boxplots of inverse Simpson diversity index for A) all vertebrate species, B) potential Humboldt marten (*Martes caurina humboldtensis*) prey species (small mammals and birds) and C) carnivores and potential competitors, detected with camera traps across 4 vegetation types in coastal Oregon.** The black lines denote the medians, *x* the average, and boxes the 25% and 50% quartiles.

Vertebrate diversity was significantly lower in interior forest compared to the coastal shrub forest (*β* = -3.85, *P* = <0.0001) (Fig 5A). Similarly, diversity of potential prey species was significantly lower in interior forest compared to the coastal shrub forest (*β* = -2.91, *P* = 0.0002) but similar to seasonally-flooded shore pine forest (*β* = -0.07, *P* = 0.999). The diversity of carnivore species was higher in coastal forests compared to interior forest (*β* = 0.634, *P* < .0001) (Fig 5C).

### Vegetation types

**Fig 6:**
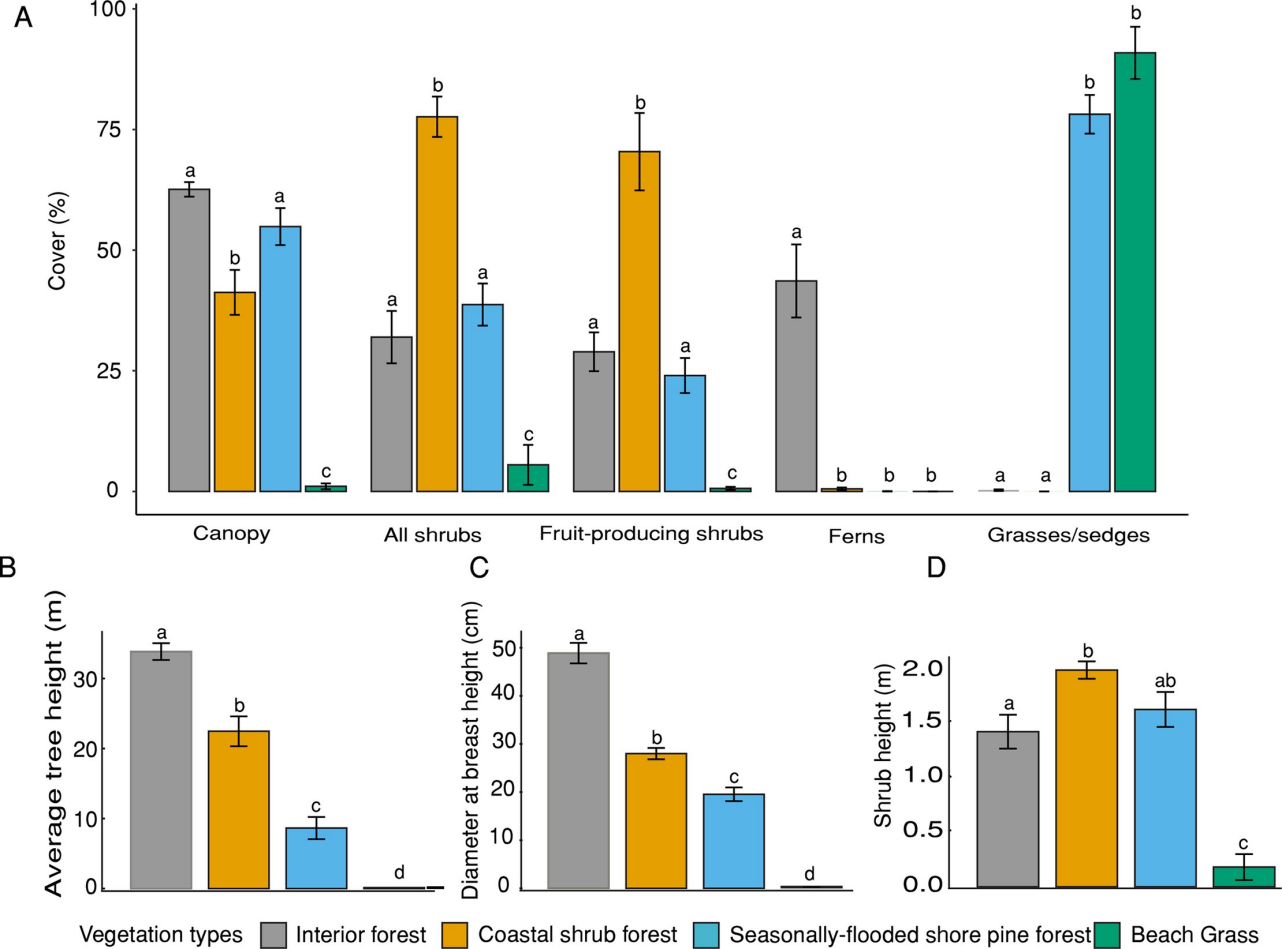
Vegetation data was collected at each prey camera station, averaged across camera stations within a grid and averaged between grids in different vegetation types (interior forest *n* = 12 grids, coastal shrub forest *n* = 7 grids, seasonally-flooded shore pine forest *n* = 8 grids, and beach grass *n* = 4 grids). We depict percent cover ($\bar{x}$ *± SE*) of the canopy, total shrub and fruit-producing shrub cover (A), B) average tree height, C) average diameter at breast height, and D) average shrub height. Different letters denote significant difference among the vegetation types after Tukey’s HSD adjustment for multiple comparisons.

Vegetation composition and cover varied among our 4 vegetation types (Fig 6). Estimated percent total shrub cover and fruit-producing shrub cover increased from beach grass to seasonally-flooded shore pine forest and reached its maximum in coastal shrub forest. Shrub cover in coastal shrub forest was significantly higher than in the interior forest (Fig 6A).

Estimated shrub height was similar between the interior forest and seasonally-flooded shore pine forest and tallest in coastal shrub forest (Fig 6D). Our estimates of canopy cover were similar between the interior and seasonally-flooded forest but lower in the coastal shrub forest (Fig 6A). Estimated average tree height (Fig 6B) and diameter (Fig 6C) were the highest in the interior forest. Understory vegetation within the 4 vegetation types were highly distinct with a few species dominating. The beach grass vegetation type consisted mainly of European beach grass (*Ammophila arenaria*) while the seasonally-flooded shore pine forest was dominated by slough sedge (*Carex obnupta*) within the understory. The interior forest understory was slightly more variable but consisted often of sword fern (*Polystichum munitum*). Dense shrub cover in the coastal shrub forest permitted little sun to reach the ground and was almost devoid of ground cover (Fig 6A). Approximate forest age of our randomly selected grids varied widely within vegetation types. The remotely sensed database suggested the seasonally-flooded shore pine community ranged 15–62 years old (*n* = 8, 37.2 ± 6.4, average ± SE), coastal shrub forest 33–70 years old (*n* = 7, 53 ± 5.7) and interior forest 23–194 years old (*n* = 12, 80.7 ± 17).

## Discussion

Although Humboldt martens in northern California have been associated with mature forests [13], our data support the conclusion of previous distribution surveys that suggest Humboldt martens in the central Oregon Coast Range currently appear to be restricted to a narrow band of young coastal forest (< 70 years) on the Oregon Dunes rather than the adjacent, older, interior forest [11]. Not only are martens seemingly absent from the historically occupied interior forest [11,44], but density estimates from the small area of adjacent young coastal forest are higher than all documented North American marten populations [13]. To explore the cause of this puzzling distribution we quantified marten diet and tested a series of plausible hypotheses. We found that 1) food availability (small vertebrate and fruit resources) was greater in the young coastal forests, 2) that potential predators, particularly bobcats, were less common in the coastal forests, 3) potential competitors were more common in coastal forests compared to interior forests, and 4) vegetation in the coastal forests provided more fruit and overhead shrub cover. This rapid assessment of potential mechanisms responsible for the apparent restricted distribution of martens was facilitated by a novel use of camera traps on replicated grids to produce an index of prey abundance and DNA metabarcoding to provide fine taxonomic resolution of marten diet.

### Marten diet

Marten diets were highly diverse but consisted primarily of 3 main categories: voles, birds and berries. The dominant consumption of voles was consistent with North American marten diet studies [33,45,46] but we detected substantially lower frequency of occurrence of squirrel species [45,47] and shrews [48]. Humboldt marten diet in northern California has been shown to heavily rely on chipmunks [49] which contrasts our study, where chipmunks were only detected in 2% of the marten scats despite being highly abundant in the coastal forests (Fig 2). Frequency of birds in the diet (53.3%) was much higher than the 10–20% typically described in North American martens’ diet [33]. Diet diversification [50] and increased bird consumption [51] has been suggested to result from low availability of voles; however, voles were abundant in the coastal forests and were consumed frequently throughout the year. High consumption of birds in this study may result from mild temperatures and seasonally high occurrence of fruits, which in combination support an abundance of resident and migratory birds as a prey resource for martens through the fall and winter. Alternatively, high bird consumption may result from increased hunting success due to structurally complex understory vegetation [26]. Dense ericaceous shrub cover in coastal forests may attract frugivorous birds by providing food and cover from avian predators. Yet, nearly impenetrable shrub layer may also reduce the ability of birds to detect predators such as martens that are actively foraging underneath the shrubs and limiting vertical escape routes [52]. In addition, ericaceous shrubs provide fruit that are readily consumed by martens, a result (also observed using stable isotope assessment of diet [53]), potentially allowing them to opportunistically prey on vertebrates while foraging for fruit. Diet of North American marten populations appear to vary [49,53], suggesting generalizing across populations may not be valid even within this subspecies.

This was the first study on the diet of a *Martes* species using DNA metabarcoding, which enabled us to present a taxonomic precision previously not achievable using mechanical sorting that earlier studies have relied on [47–49] but see [54]. Avian resolution in the diet was previously restricted to ‘birds’ [33,47] or ‘small birds’ versus ‘large birds’ [48,49]. Here, we were able to demonstrate a broad variety of passerines consumed. Importantly, shorebirds including the Federally Threatened snowy plover (*Charadrius nivosus*) were not identified as consumed, tentatively suggesting martens were not a threat to this species which is associated with open beaches (see also [53]).

### Prey abundance

We found evidence of increased food resources in the coastal shrub forest and the seasonally-flooded shore pine forest compared to the interior forest. In particular, voles, deer mice and fruit, which were all highly represented in marten diets, were significantly more abundant in coastal shrub forests. While previous studies have found a positive or neutral association between small mammals and mature forests [55–57], our findings suggest that younger coastal forests had a higher relative abundance of several small mammal species (Fig 2). Increased forest age and small mammal abundance has been positively associated with fungi and fruit, known food sources for many small mammals [58,59]. During our study, the coastal shrub forest contained more fruit-producing shrubs, and although not investigated here, potentially increased fungi. Ashkannejhad and Horton [59] found a higher diversity of fungi in coastal forests compared to interior forest, including species commonly consumed by small mammals [55,60]. In addition, highly abundant ericaceous species present in coastal forests such as salal, Pacific rhododendron (*Rhododendron macrophyllum*) and huckleberries (*Vaccinium* spp), host a range of ectomycorrhizal fungi [61] potentially supplying small mammals with fungi in addition to fruit compared to interior forest. Thus, mechanisms for increased small mammal abundance in our study may be correlated with several factors: 1) abundant fungi present in coastal forests, 2) small mammal species in our study area were supported by fruits and seeds from high relative abundance of fruit-producing shrubs, or 3) these small mammal species are top-down limited, and predator protection in the form of a dense shrub layer has resulted in increased prey abundance.

Bird abundance was higher in coastal forests compared to interior forest. Fox sparrows, song sparrows, spotted towhees, hermit thrushes (*Catharus guttatus*) and Steller’s jays (*Cyanocitta stelleri*) were significantly more abundant in the coastal forests, but of these, only Steller’s jays were consumed by martens during our study (Table 1). Varied thrush was the most frequently consumed prey species by marten and abundant in all forests. In addition, martens preyed upon several bird species that we failed to detect with our camera-based surveys such as yellow-rumped warblers (*Setophaga coronata*), American coot (*Fulica americana*), and golden-crowned kinglets (*Regulus satrapa*). Failure to detect these species with our cameras may reflect the bird’s ecology (aquatic vs terrestrial habitat) or small body size, which could limit detectability [62]. It is likely that the fruit-producing shrubs (e.g., huckleberries, salal) attract frugivorous birds [63] which may explain the higher abundance of some birds in the coastal forests (Fig 3). Whatever the mechanism, our hypothesis of increased prey availability in the coastal forest was supported and remains a viable explanation for determining the observed distribution of martens.

### Competition and predation

Gray foxes, raccoons, and western spotted skunks have an omnivorous diet and may include diet items similar to martens [34], potentially making these species competitors for nutritional resources. Our camera-based surveys revealed that gray foxes and raccoons were, like martens, detected almost exclusively in the coastal forests. This observation suggests that release from competition with these species was not a plausible hypothesis for why martens appear to be restricted to the coastal forests. Nonetheless, western spotted skunks, the most similar species in terms of body size, phylogeny, and small cavity utilization, were relatively rare in the coastal forests and common in the interior forest. It is thus plausible that marten face some degree of competition with spotted skunks in the interior forest, although unlikely because the extent of diet overlap is unknown, they have divergent diel activity patterns, spotted skunks are smaller-bodied, and there is no evidence of interference competition between these species. In addition, we could not dismiss the competitive release hypothesis as similar bodied avian competitors were not surveyed for but could provide substantial competition for martens in addition to being their direct predators [19].

Bobcats, an important mammalian predator of martens [19,64], were significantly more common in the interior forest. The extensive shrub cover in the coastal shrub forest is likely to facilitate escape from larger-bodied bobcats and provide overhead cover to avoid avian predation. However, martens were frequently detected in the seasonally-flooded shore pine forest, where sedges provide some understory structure, but shrub cover was not distinct from the interior forest. Although shrub cover was lower in seasonally-flooded shore pine forest, seasonal flooding may have contributed to the relative absence of bobcats and other competitors there, perhaps conferring some competitive advantage to the scansorial marten if they are able to travel through the canopy more readily when forests are flooded (Fig 6). Although shrubs were not prevalent in all forests used by martens, other studies in the Pacific states have shown strong association with shrub cover and therefore further mechanistic studies examining whether shrubs provide protective cover may be warranted [12,65].

### Rapid community assessment for imperiled species

The causal mechanisms contributing to the rarity of species are often challenging to discern, and manipulative experiments are often infeasible or unethical. In addition, when conservation measures are urgently needed, as is the case with Humboldt martens in Oregon, a framework for rapid identification of potential stressors limiting population growth and spread is warranted. The conservation of most species is hampered by a general lack of natural history and community ecology information. This includes limited information about distribution, abundance, and diet of species across trophic levels. For example, in our study area, we had no information on what martens were eating, relative abundance of their prey, potential predators and competitors across vegetation types, and we still lack basic information on avian predators and competitors, and cause-specific mortality of martens. In addition, we are deficient in basic information about the base of the food web. For instance, small mammals are a basal resource, but the degree to which each species is energetically supported by fungi, lichen, fruit, seeds, or insects is poorly known. The lack of information about seasonally important prey and drivers of prey abundance limits our ability to predict how differences in vegetation affect marten populations.

We benefited from new technologies, such as DNA metabarcoding, and the availability of affordable and efficient camera traps to broadly survey the vertebrate community. By using replicated and distinct camera survey methods, we provided insights into biases present when using only 1 set or bait type, especially if the goal is to broadly describe the vertebrate community. For instance, marten detections were strongly associated with stations baited with chicken and lure, whereas bobcats were primarily detected on unbaited trail cameras (Table 3). Gray foxes, raccoons, short-tailed weasels (*Mustela erminea*), opossums (*Didelphis virginiana*), and spotted skunks were detected more frequently at our small mammal grids baited with oats, seeds, nuts, and jams (Table 3) than at carnivore cameras. Our study was not designed to evaluate survey efficiency, but differing bait types and camera density per sample unit (i.e., 1 vs. 20 cameras in a grid) allowed us to have a higher probability of detecting a suite of vertebrate species.

Our approach to producing an index of abundance assumed that animals are detected more frequently when abundant. Although this is largely self-evident, if detectability of animals was highly variable across vegetation types for other reasons, such as behavioral changes that result in differential attraction to baits, then our inference about abundance may be biased. For example, if voles were consistently less willing to approach bait in the interior forest and trigger the camera, then our inference that they were less abundant there is not necessarily robust. Although unlikely, this is an important caveat, but more invasive methods to estimate abundance, such as mark-recapture, have their own limitations. In particular, such approaches are taxonomically limited to certain species, whereas cameras detect a suite of species including birds and mammals across a range of body sizes from small shrews to large-bodied carnivores. This taxonomic limitation is key when assessing the prey base of a species with such a diverse diet. In addition, mark-recapture approaches are time-consuming, and it is logistically challenging to implement high levels of replication on landscape scales. With these considerations in mind, the use of these rapid multi-species methods can be a valuable tool for the conservation of imperiled or relatively unknown species.

### Conclusion

Our results provide insight into the importance of complex and cohesive understory vegetation, which provide protection and food availability for marten and their prey. We found a higher relative prey abundance and fewer detections of bobcats in the coastal forests compared to the interior forest. Several small-bodied carnivores were common in the coastal forests, therefore release from competition from terrestrial competitors did not seem a plausible explanation for the restricted distribution of martens, but we could not dismiss avian competition as we did not adequately survey for raptors.

A potential explanation for the absence of martens in the interior forest is that the mature forest of the Siuslaw National Forest, much of which was removed by fires in the 19^th^ century and timber harvest in the 20^th^ century, may not yet be old enough to provide the complex understory and decay related features, often used for resting and denning [23,66]. Complex understories are characteristic of very old forests in the Pacific Northwest (e.g. > 300 years), which contain extensive downed wood and shrub cover in long-lived tree-fall gaps [22]. Our results suggest that direct and indirect effects of understory shrubs may play a key role in the distribution of martens. Fruit-producing shrubs provide a direct nutritional resource that was a key component of marten diets, and dense shrub cover can reduce predation risk from larger mammalian predators that cannot navigate through shrubs and avian predators that cannot access the area beneath the shrub layer.

Based on our data and literature on marten movement [67], maintaining connected forests within the Oregon Dunes National Recreation Area in conjunction with habitat restoration to promote recruitment of a dense, tall fruit-producing shrub layer within interior forest would likely benefit Humboldt marten populations. Our study was limited to publicly owned forests leaving some private landholdings unstudied. Future survey efforts could focus on filling in these gaps to more evenly cover the interior forest. In addition, further studies to facilitate marten conservation could include identification of structures used as rest and den sites within the young coastal forests, causes of mortality, threats of disease and rodenticides, and opportunities for dispersal into the interior forest [68]. Based on historic records and knowledge of marten habitat elsewhere [44], we know that parts of the interior forest were once occupied by martens and have the potential to be occupied again if conditions improve. If the fruit-producing shrubs in the coastal forests are increasing prey diversity and abundance, or reducing predation risk for martens (and their prey), future restoration efforts in the interior forest could focus on fruit-producing shrub recruitment of similar density and height to coastal shrub forests.

## Supporting information

S1 Document Humboldt marten (*Martes caurina humboldtensis*) scat collection methods. (DOCX)

S2 Document Potential Humboldt marten (*Martes caurina humboldtensis*) prey species sequencing methods. (DOCX)

S3 Document R code for all camera and vegetation data analyses during Humboldt marten (*Martes caurina humboldtensis*) study in the central Oregon coast. (DOCX)

S1 Fig Study area depiction and examples of photos obtained with remote cameras across our four vegetation types during a study of Humboldt marten (*Martes caurina humboldtensis*) occurrence in the central Oregon coast: A) Beach grass, B) Seasonally-flooded shore pine forest, C) Coastal shrub forest, D) Interior forest.
(DOCX)

S1 Table List of all species detected with camera traps in four vegetation types during a study of Humboldt marten (*Martes caurina humboldtensis*) distribution. We combined data into 4 vegetation types: Beach grass, Seasonally-flooded shore pine forest, Coastal shrub forest and Interior forest. We depict the number of photographs obtained for each species type.
(DOCX)
