# Supplementary material for: Biotic factors influencing the unexpected distribution of a Humboldt marten (Martes caurina humboldtensis) population in a young coastal forest
Source: PLoS One. 2019 May 1;14(5):e0214653. doi: 10.1371/journal.pone.0214653 (PMC6493723; doi:10.1371/journal.pone.0214653)
Supplement: S1 Document — (DOCX) [file pone.0214653.s001.docx]

**Document S1: Marten scat collection methods**

We collected scats during a study of Humboldt marten (*Martes caurina humboldtensis*) occurrence and natural history. Approximately 1/3 of our samples were collected by scent detection dog teams from University of Washington’s Center for Conservation Biology’s Conservation Canines program (see [1] for details). The teams conducted time-constrained searches at randomly selected 3x3 km sample units to identify marten distribution and range, surveying each sample unit for at least four hours. We assumed this would allow adequate effort to locate scats. Scent detection teams consisted of handler and dog with a minimum of 480 hours of training using lab and simulated field trails (e.g., hidden scats on training boards and in the duff). Maintaining dog focus for rare species can be increased by having multiple targets, allowing a reward for any of the target species. Although we only report marten results, the teams were instructed to collect scat from marten, bobcat (*Lynx rufus*), mountain lion (*Puma concolor*), fisher (*Pekania pennanti*), and porcupine (*Erithrozon dorsatum*) so we could gain information on potential competitors, predators, and other species of interest. Surveys occurred during three periods: 25 Apr-07 May 2015 (n = 10 sample units), 30-31 March 2016 (at 6 marten rest locations), 23 Jul-12 Aug 2017 (n = 37 sample units).

We collected marten scats at known marten rest sites and from trap locations (October 2015-March 2016, see [2] for detailed methods). Rest structures were located opportunistically weekly using telemetry homing procedures. All capture and handling procedures were approved by the USDA Forest Service’s Institute for Animal Care and Use Committee (USFS 2015-002) under an Oregon Department of Fish and Wildlife Scientific Take Permit (ODFW 119-15).

**References**

1. Moriarty KM, Linnell MA, Thornton JE, Watts GW. Seeking efficiency with carnivore survey methods: A case study with elusive martens. Wildl Soc Bull. 2018;42(3):403–13.

2. Moriarty KM, Linnell MA, Chasco BE, Epps CW, Zielinski WJ. Using high-resolution short-term location data to describe territoriality in Pacific martens. J Mammal. 2017;98(3):679–89.
