# Supplementary material for: Biotic factors influencing the unexpected distribution of a Humboldt marten (Martes caurina humboldtensis) population in a young coastal forest
Source: PLoS One. 2019 May 1;14(5):e0214653. doi: 10.1371/journal.pone.0214653 (PMC6493723; doi:10.1371/journal.pone.0214653)
Supplement: S2 Document — (DOCX) [file pone.0214653.s002.docx]

**Document S2: Potential Humboldt marten (*Martes caurina humboldtensis*) prey species sequencing methods**

We created a 12S reference library for vertebrate species not present in Genbank to improve taxonomic identification for species in Oregon, USA. Approximately 25 mg of tissue was removed from local frozen specimens and preserved in 100% ethanol. We extracted DNA using the Qiagen Blood & Tissue kit following the manufacturer’s instructions. The DNA extracts were amplified using the primer pair 12SeqF (Allen, unpublished data) and H1259 (1). We performed PCRs in a total volume of 20 μL using the following reagent mixtures: 10 μL AmpliTaq Gold 360 Master Mix (Life Technologies), 0.2 μL of each primer (200 Nm final concentration), 0.2 μL BSA (0.5 mg/ml final concentration), 8.4 μL of water, and 1 μL final DNA extract elution (including extraction controls and a PCR blank). Following a 10 min, initial denaturation at 95°C, the cycling conditions were: 39 cycles of 95°C for 30 seconds, 58°C for 30 seconds, 72°C for 1 min, and a final extension at 72°C for 7 min. We purified the post-PCR products using ExoSAP-IT PCR Clean-up Kit (USB) before sending the samples for Sanger sequencing at the Center for Genome Research and Biocomputing, Oregon State University.

**References**

1. Kitano T, Umetsu K, Tian W, Osawa M. Two universal primer sets for species identification among vertebrates. Int J Legal Med. 2007;121(5):423–7.
