# Supplementary material for: Biotic factors influencing the unexpected distribution of a Humboldt marten (Martes caurina humboldtensis) population in a young coastal forest
Source: PLoS One. 2019 May 1;14(5):e0214653. doi: 10.1371/journal.pone.0214653 (PMC6493723; doi:10.1371/journal.pone.0214653)
Supplement: S3 Document — (DOCX) [file pone.0214653.s003.docx]

**S3 Document: R code for all camera and vegetation data analyses**

library(chron)

library(plyr)

library(dplyr)

library(tidyr)

library(ggplot2)

library(grid)

library(gridExtra)

library(lme4)

library(car)

library(nlme)

library(lme4)

library(vegan)

library(reshape2)

library(tidyr)

library(grid)

library(gmodels)

library(lsmeans)

library(FSA)

library(dunn.test)

library(car)

library(reshape2)

#Set working directory

###############################

#Read in the data (Bird species were analyzed separately with identical code)

#Vegetation community names are different than in the paper:

#Ericaceous_Forest = Coastal shrub forest

#Deflation_Plain = Seasonally-flooded shore pine forest

###############################

All_Data=read.csv("Mammal_cameradata.csv",header=TRUE)

#use the as.Date( ) function to convert character data to dates.

All_Data$Date_of_photos=as.Date(All_Data$Date_of_photos, "%m/%d/%Y")

All_Data$Start_date=as.Date(All_Data$Start_date, "%m/%d/%Y")

All_Data$End_date=as.Date(All_Data$End_date, "%m/%d/%Y")

species<-c("APRU","GLSA", "NECI", "OTBE", "PEMA", "RARA", "SOSP", "SYBA", "TADO", "TATO", "VOLE")

# Create Camera_day variable

# Create the cumulative sum of each species

Cumulativedata=All_Data %>% group_by(Camera_ID) %>%

mutate(Camera_day=Date_of_photos - Start_date+1) %>%

mutate(Total_days=End_date - Start_date) %>%

mutate_at(species,funs(cumsum))

#Summarise the maximum cumulative data of each species per camera per camera day

Max_Cum_Pics=Cumulativedata %>% group_by(Vegetation_Type,Site_ID,Camera_ID,Camera_day) %>% summarise_at(species,funs(max))

#Make the data binomial

PA_Cum_Pics=data.frame(Max_Cum_Pics)

for(i in 5:15){

PA_Cum_Pics[,i]=pmin(PA_Cum_Pics[,i],1)

}

###############################

#Create Small mammal grid set (cameras labelled A-T) - Remove the Carnivore(V) and Trail cameras(U)

###############################

MammalSet=which(substr(PA_Cum_Pics$Camera_ID,10,10)!="U"&substr(PA_Cum_Pics$Camera_ID,10,10)!="V")

#Produce subset of small mammal data

PA_smammal=PA_Cum_Pics[MammalSet,]

#Summarise the binomial data for each species per vegetation type, site (grid) and camera day

Site_PA_Summary=PA_smammal %>% group_by(Vegetation_Type,Site_ID,Camera_day) %>% summarise_at(species,funs(sum))

#Summary of small mammal data on camera day 7 and day 1

Site_PA_Summary_Day7=Site_PA_Summary[Site_PA_Summary$Camera_day==7,]

Site_PA_Summary_Day1=Site_PA_Summary[Site_PA_Summary$Camera_day==1,]

#Select the species columns

#yes = # of cameras that detected the species

#no = # of cameras that did not detect the species

#Day 7

yes=Site_PA_Summary_Day7[,(4:14)]

no=20-yes

# selecting all species except deer mouse

Prey_Day7<-Site_PA_Summary_Day7[,c(1:7, 9:14)]

#Day 1 - Specifically for deer mice to avoid detection saturation at all cameras

yes1=Site_PA_Summary_Day1[,4:14]

no1=20-yes1

#Select PEMA

PEMA_Day1<-(Site_PA_Summary_Day1[, 8])

#Add PEMA (deer mouse) to the main dataframe

Prey_diversity<-cbind(as.data.frame(Prey_Day7),as.data.frame(PEMA_Day1))

############################### Species diversity #############################################

cols <- c(4:14)

Only_preyspp<-(Prey_diversity[,cols])

###Species Richness###

Prey_richness <- specnumber(Only_preyspp)

Prey_diversity$Prey_richness<-with(Prey_diversity,Prey_richness)

Prey_richness_mean<-ddply(Prey_diversity,.(Vegetation_Type), summarize,

mean=mean(Prey_richness),

sd=sd(Prey_richness),

s=sd(Prey_richness)/sqrt(NROW(Prey_richness)),

lower.ci=mean(Prey_richness)-((sd*1.96)/sqrt(NROW(Prey_richness))),

upper.ci=mean(Prey_richness)+((sd*1.96)/sqrt(NROW(Prey_richness))),

n=NROW(Prey_richness))

#Inverse Simpson

Invsimpson_prey<-diversity(Only_preyspp, index = "invsimpson", MARGIN = 1)

Prey_diversity$Invsimpson_prey<-with(Prey_diversity,Invsimpson_prey)

#Prey boxplot

(Prey_Diversity_box <- ggplot(Prey_diversity, aes(Vegetation_Type, Invsimpson_prey),reorder(factor(Vegetation_Type),factor(Vegetation_Type),length))+

geom_boxplot(outlier.size = -1)+

xlab("Vegetation Type")+

theme(axis.title.y=element_blank())+

theme_bw(15)+theme(panel.grid.minor=element_blank(),panel.grid.major=element_blank())+

stat_summary(fun.y=mean,shape=4, size = 2, col='black', geom='point')+theme(axis.title.y=element_blank())+scale_x_discrete(breaks=c("Interior_Forest", "Ericaceae_Forest", "Deflation_Plain", "Beach_Grass"), labels=c("Interior Forest", "Coastal Shrub Forest", "Seasonally-flooded Lodgepole Forest", "Beach Grass")) )

############################## Index of relative abundance #################################

#Convert data into long form

Prey_Day7_long<-Prey_diversity %>% gather(Species, Abundance, APRU:PEMA)

#Relativise abundance for each species per site

#1 Group species per site since 3 sites had less than 20 functioning cameras

Prey_Day7_sites<-ddply(Prey_Day7_long,.(Vegetation_Type, Site_ID))

#2 Subset sites depending on # of cameras

Sites_20<-(Prey_Day7_sites[c(1:44, 56:121, 133:242, 254:297, 309:319), ])

Sites_19<-(Prey_Day7_sites[c(45:55, 243:253, 298:308, 320:341), ])

Sites_17<-(Prey_Day7_sites[c(122:132), ])

#Relativise each species per site

Sites_20["Prop"]<- NA

Sites_20$Prop <- Sites_20$Abundance/20

Sites_19["Prop"]<- NA

Sites_19$Prop <- Sites_19$Abundance/19

Sites_17["Prop"]<- NA

Sites_17$Prop <- Sites_17$Abundance/17

#Bind the different sites back together

Prop_abundance<-rbind(Sites_20,Sites_19,Sites_17)

#Calculate mean and SE based on proportion

Prey_Day7_mean<-ddply(Prop_abundance,.(Vegetation_Type, Species), summarize,

mean=mean(Prop),

sd=sd(Prop),

s=sd(Prop)/sqrt(NROW(Prop)),

lower.ci=mean(Prop)-((sd*1.96)/sqrt(NROW(Prop))),

upper.ci=mean(Prop)+((sd*1.96)/sqrt(NROW(Prop))),

n=NROW(Prop))

##################################### GLMER ##############################################

Site_PA_Summary_Day7new=data.frame(Site_PA_Summary_Day7,yes,no) #For all species except deer mouse

Site_PA_Summary_Day1new=data.frame(Site_PA_Summary_Day1,yes1,no1) #For deer mouse

#Using VOLE as an example

VOLE.mod1<-glmer(cbind(yes$VOLE,no$VOLE)~Vegetation_Type+(1|Site_ID),data=Site_PA_Summary_Day7new, family="binomial")

summary(VOLE.mod1)

VOLElsm <- lsmeans(VOLE.mod1, ~ Vegetation_Type)

summary(VOLElsm, type = "response")

summary(pairs(VOLElsm), type = "response")

####################################################################################################################################

#Carnivore analysis

Master_CarnivoreData_sites=read.csv("Master_Carnivore_Data_sites.csv",header=TRUE)

#############################

#Convert data into long form

#############################

Carnivores_long_sites<-Master_CarnivoreData_sites %>% gather(Species, Abundance, DIVI:URCI)

############################

#Create Index of abundance

############################

Carnivores_long_sites$Total_days <- as.numeric(as.character(Carnivores_long_sites$Total_days))

Carnivores_long_sites["A_index"]<- NA

Carnivores_long_sites$A_index <- Carnivores_long_sites$Abundance / Carnivores_long_sites$Total_days

####################################

#Days detected (Yes) and days not detected (No)

####################################

Carnivores_long_sites["Yes"]<- NA

Carnivores_long_sites$Yes <- Carnivores_long_sites$Abundance

Carnivores_long_sites["No"]<- NA

Carnivores_long_sites$No <- Carnivores_long_sites$Total_days - Carnivores_long_sites$Yes

spnames<-unique(Carnivores_long_sites$Species)

for (name in spnames){

print(paste("This regression is for ", name))

print(summary(glmer(cbind(Yes, No) ~ Vegetation_Type + Survey_Type3 + Cam_Type +(1 | Site_ID), data = subset(Carnivores_long_sites, Species == name), family="binomial")))

coords = cbind(subset(Carnivores_long_sites, Species == name)$y, subset(Carnivores_long_sites, Species == name)$x)

knn = knn2nb(knearneigh(coords, k=1, longlat=FALSE), sym=TRUE)

print(Moran.I(residuals(glmer(cbind(Yes, No) ~ Vegetation_Type + Survey_Type3 + Cam_Type + (1 | Site_ID), data = subset(Carnivores_long_sites, Species == name), family="binomial")), dists.inv))

sp_lag_c = lag.listw(nb2listw(knn,style="B"), residuals(glmer(cbind(Yes, No) ~ Vegetation_Type + Survey_Type3 + Cam_Type + (1 | Site_ID), data = subset(Carnivores_long_sites, Species == name), family="binomial")))

print(paste("This SPATIAL regression is for ", name))

print(summary(glmer(cbind(Yes, No) ~ Vegetation_Type + Survey_Type3 + Cam_Type + sp_lag_c + (1 | Site_ID), data = subset(Carnivores_long_sites, Species == name), family="binomial")))

print(Moran.I(residuals(glmer(cbind(Yes, No) ~ Vegetation_Type + Survey_Type3 + Cam_Type + sp_lag_c + (1 | Site_ID), data = subset(Carnivores_long_sites, Species == name), family="binomial")), dists.inv))

}

dists=as.matrix(dist(subset(subset(Carnivores_long_sites, Species == name)[,9:10])))

dists.inv=1/dists

diag(dists.inv)=0

dists.inv[dists.inv==Inf]=0

w=dists.inv

lw <- mat2listw(w)

lwW <- nb2listw(lw$neighbours, glist=lw$weights, style="W")

#######################################################################################################################################

#Vegetation data

library(FSA)

library(dunn.test)

Veg_site_level=read.csv("Veg_site_level.csv",header=TRUE)

#Make data frame into long form

Veg_melted<-melt(Veg_site_level)

#Relativise all proportion data

Canopy_subset<-Veg_melted[1:217,]

Canopy_subset$Prop <- Canopy_subset$value/100

Veg_tree_HTM<-Veg_melted[218:279,]

Shrub_subset<-Veg_melted[280:868,]

Shrub_subset$Prop <- Shrub_subset$value/100

Veg_Shrub_HTM<-Veg_melted[869:899,]

Grass_ferns_subset<-Veg_melted[900:961,]

Grass_ferns_subset$Prop <- Grass_ferns_subset$value/100

Shrub_Cover<-Veg_melted[962:992,]

Shrub_Cover$Prop <- Shrub_Cover$value/131

Canopy_Cover<-Veg_melted[993:1023,]

Canopy_Cover$Prop <- Canopy_Cover$value/100

Fruit_Cover<-Veg_melted[1024:1054,]

Fruit_Cover$Prop <- Fruit_Cover$value/112.75

AVETREEDIA<-Veg_melted[218:248,]

#Bind the data frames

Veg_prop_data<-rbind(Canopy_subset, Shrub_subset, Grass_ferns_subset, Shrub_Cover, Canopy_Cover, Fruit_Cover)

Veg_HTM_data<-rbind(Veg_tree_HTM, Veg_Shrub_HTM, AVETREEDIA)

#P-values

#Using diameter at breast height as an example

Dbh<-subset(Veg_HTM_data,variable=="AVETREEDIA")

kruskal.test(value ~ Vegetation_Type, data = Dbh)

Summarize(value ~ Vegetation_Type, data = Dbh)

PT = dunnTest(value ~ Vegetation_Type,data=Dbh, method="bh")
