# Supplementary material for: Biotic factors influencing the unexpected distribution of a Humboldt marten (Martes caurina humboldtensis) population in a young coastal forest
Source: PLoS One. 2019 May 1;14(5):e0214653. doi: 10.1371/journal.pone.0214653 (PMC6493723; doi:10.1371/journal.pone.0214653)
Supplement: S1 Fig — Study area depiction and examples of photos obtained with remote cameras across our four vegetation types during a study of Humboldt marten (Martes caurina humboldtensis) occurrence in the central Oregon coast: A) Beach grass, B) Seasonally-flooded shore pine forest, C) Coastal shrub forest, D) Interior forest. (DOCX) [file pone.0214653.s004.docx]

**Figure S1: Study area depiction and examples of photos obtained with remote cameras across our four vegetation types during a study of Humboldt marten (*Martes caurina humboldtensis*) occurrence in the central Oregon coast: A) Beach grass, B) Seasonally-flooded shore pine forest, C) Coastal shrub forest, D) Interior forest.**

**Figure S1: Examples of photos obtained with remote cameras across the four vegetation types**: A) Beach grass, B) Seasonally-flooded shore pine forest, C) Coastal shrub forest, D) Interior forest. Top row (left-right): California ground squirrel (*Otospermophilus beecheyi*), Gray fox (*Urocyon cinereoargenteus*), Humboldt marten (*Martes caurina humboldtensis*), Western spotted skunk (*Spilogale gracilis*). Middle row: Short-tailed weasel (*Mustela erminea*), Vole spp. (*Arvicolinae* spp), Steller's jay (*Cyanocitta stelleri*), Douglas squirrel (*Tamiasciurus douglasii*). Bottom row: Deer mouse (*Peromyscus maniculatus*), Raccoon (*Procyon lotor*), Townsend's chipmunk (*Tamias townsendii*), Virginia opossum (*Didelphis virginiana*).
