# Supplementary material for: Biotic factors influencing the unexpected distribution of a Humboldt marten (Martes caurina humboldtensis) population in a young coastal forest
Source: PLoS One. 2019 May 1;14(5):e0214653. doi: 10.1371/journal.pone.0214653 (PMC6493723; doi:10.1371/journal.pone.0214653)
Supplement: S1 Table — We combined data into 4 vegetation types: Beach grass, Seasonally-flooded shore pine forest, Coastal shrub forest and Interior forest. We depict the number of photographs obtained for each species type. (DOCX) [file pone.0214653.s005.docx]

**Table S1*:* List of all species detected with camera traps in four vegetation types during a study of Humboldt marten (*Martes caurina humboldtensis*) distribution. We combined data into 4 vegetation types: Beach grass, Seasonally-flooded shore pine forest, Coastal shrub forest and Interior forest. We depict the number of photographs obtained for each species per vegetation type.**

| **Species** | **Common name** | **Beach grass** | **Seasonally-flooded shore pine forest** | **Coastal** | **Interior** |
| --- | --- | --- | --- | --- | --- |
|  |  |  |  | **shrub forest** | **forest** |
| **Mammals** |  |  |  |  |  |
| CARNIVORA |  |  |  |  |  |
| *Lynx rufus* | Bobcat | 0 | 24 | 0 | 64 |
| *Martes caurina humboldtensis* | Humboldt marten | 0 | 287 | 458 | 0 |
| *Mustela erminea* | Short-tailed weasel | 15 | 15 | 54 | 30 |
| *Mustela frenata* | Long-tailed weasel | 12 | 3 | 3 | 6 |
| *Neovision vision* | American mink | 0 | 3 | 3 | 0 |
| *Procyon lotor* | Raccoon | 0 | 2253 | 704 | 11 |
| *Puma concolor* | Mountain lion | 0 | 0 | 0 | 12 |
| *Spilogale gracilis* | Western spotted skunk | 0 | 0 | 721 | 762 |
| *Urocyon cinereoargenteus* | Gray fox | 0 | 1049 | 458 | 0 |
| *Ursus americanus* | American black bear | 0 | 55 | 274 | 65 |
| CETARTIODACTYLA |  |  |  |  |  |
| *Cervus canadensis* | Elk | 0 | 0 | 0 | 9 |
| *Odocoileus hemionus* | Mule deer | 36 | 576 | 36 | 297 |
| DIDELPHIMORPHA |  |  |  |  |  |
| *Didelphis virginiana* | Virginia opossum | 0 | 1014 | 1141 | 11404 |
| LAGOMORPHA |  |  |  |  |  |
| *Sylvilagus bachmani* | Brush rabbit | 360 | 972 | 1502 | 252 |
| SORICOMORPHA |  |  |  |  |  |
| *Sorex species* | Shrew species | 63 | 3915 | 5977 | 5134 |
| RODENTIA |  |  |  |  |  |
| *Aplodontia rufa* | Mountain beaver | 0 | 0 | 0 | 105 |
| *Arvicolinae species* | Vole species | 1296 | 1330 | 2378 | 806 |
| *Castor canadensis* | North american beaver | 0 | 51 | 0 | 0 |
| *Erethizon dorsatum* | North American porcupine | 30 | 24 | 0 | 0 |
| *Glaucomys oregonensis* | Northern flying squirrel | 0 | 3 | 3907 | 875 |
| *Neotoma cinerea* | Bushy-tailed wood rat |  |  |  |  |
| *Otospermophilus beecheyi* | California ground squirrel | 477 | 0 | 0 | 0 |
| *Peromyscus maniculatus* | Deer mouse | 55861 | 136221 | 172125 | 198449 |
| *Rattus rattus* | Black rat |  |  | 3 | 39 |
| *Tamias townsendii* | Townsend’s chipmunk | 0 | 235 | 12703 | 2170 |
| *Tamiasciurus douglasii* | Douglas squirrel | 3 | 335 | 636 | 1045 |
| **Birds** |  |  |  |  |  |
| CHARADRIIFORMES |  |  |  |  |  |
| *Gallinago delicata* | Willson's snipe | 3 | 39 | 0 | 0 |
| GALLIFORMES |  |  |  |  |  |
| *Bonasa umbellus* | Ruffed grouse | 0 | 0 | 0 | 18 |
| GRUIFORMES |  |  |  |  |  |
| *Rallus limicola* | Virginia rail | 0 | 9 | 0 | 0 |
| PASSERIFORMES |  |  |  |  |  |
| *Catharus guttatus* | Hermit thrush | 0 | 258 | 230 | 45 |
| *Chamaea fasciata* | Wrentit | 0 | 30 | 33 | 382 |
| *Corvus corax* | Common raven | 588 | 0 | 0 | 0 |
| *Cyanocitta stelleri* | Steller's jay | 0 | 1193 | 3473 | 39 |
| *Ixoreus naevius* | Varied thrush | 3 | 1396 | 364 | 503 |
| *Junco hyemalis* | Dark-eyed junco | 0 | 705 | 3 | 6 |
| *Melospiza melodia* | Song sparrow | 131 | 1678 | 1094 | 30 |
| *Passerella iliaca* | Fox sparrow | 0 | 1035 | 5326 | 793 |
| *Perisoreus canadensis* | Gray jay | 0 | 0 | 0 | 2363 |
| *Pipilo maculatus* | Spotted towhee | 33 | 135 | 1174 | 65 |
| *Regulus satrapa* | Golden-crowned kinglets | 0 | 0 | 0 | 6 |
| *Sturnella neglecta* | Western meadowlark | 6 | 0 | 0 | 0 |
| *Troglodytes pacificus* | Pacific wren | 0 | 39 | 83 | 380 |
| *Turdus migratorius* | American robin | 0 | 51 | 302 | 6 |
| *Zonotrichia atricapilla* | Golden-crowned sparrow | 28 | 0 | 0 | 0 |
